# TLR9 Activation Dampens the Early Inflammatory Response to *Paracoccidioides brasiliensis*, Impacting Host Survival

**DOI:** 10.1371/journal.pntd.0002317

**Published:** 2013-07-25

**Authors:** João Filipe Menino, Margarida Saraiva, Ana G. Gomes-Alves, Diogo Lobo-Silva, Mark Sturme, Jéssica Gomes-Rezende, Ana Laura Saraiva, Gustavo H. Goldman, Cristina Cunha, Agostinho Carvalho, Luigina Romani, Jorge Pedrosa, António Gil Castro, Fernando Rodrigues

**Affiliations:** 1 Life and Health Sciences Research Institute (ICVS), School of Health Sciences, University of Minho, Braga, Portugal; 2 ICVS/3B's - PT Government Associate Laboratory, Braga/Guimarães, Portugal; 3 EUVG - Escola Universitária Vasco da Gama, Castelo Viegas, Coimbra, Portugal; 4 Laboratório Nacional de Ciência e Tecnologia do Bioetanol – CTBE, Campinas, São Paulo, Brazil, and Faculdade de Ciências Farmacêuticas de Ribeirão Preto, Universidade de São Paulo, São Paulo, Brasil; 5 Department of Experimental Medicine and Biochemical Sciences, University of Perugia, Perugia, Italy; University of California San Diego School of Medicine, United States of America

## Abstract

**Background:**

*Paracoccidioides brasiliensis* causes paracoccidioidomycosis, one of the most prevalent systemic mycosis in Latin America. Thus, understanding the characteristics of the protective immune response to *P. brasiliensis* is of interest, as it may reveal targets for disease control. The initiation of the immune response relies on the activation of pattern recognition receptors, among which are TLRs. Both TLR2 and TLR4 have been implicated in the recognition of *P. brasiliensis* and regulation of the immune response. However, the role of TLR9 during the infection by this fungus remains unclear.

**Methodology/Principal findings:**

We used *in vitro* and *in vivo* models of infection by *P. brasiliensis*, comparing wild type and TLR9 deficient (^−/−^) mice, to assess the contribution of TLR9 on cytokine induction, phagocytosis and outcome of infection. We show that TLR9 recognizes either the yeast form or DNA from *P. brasiliensis* by stimulating the expression/production of pro-inflammatory cytokines by bone marrow derived macrophages, also increasing their phagocytic ability. We further show that TLR9 plays a protective role early after intravenous infection with *P. brasiliensis*, as infected TLR9^−/−^ mice died at higher rate during the first 48 hours post infection than wild type mice. Moreover, TLR9^−/−^ mice presented tissue damage and increased expression of several cytokines, such as TNF-α and IL-6. The increased pattern of cytokine expression was also observed during intraperitoneal infection of TLR9^−/−^ mice, with enhanced recruitment of neutrophils. The phenotype of TLR9^−/−^ hosts observed during the early stages of *P. brasiliensis* infection was reverted upon a transient, 48 hours post-infection, neutrophil depletion.

**Conclusions/Significance:**

Our results suggest that TLR9 activation plays an early protective role against *P. brasiliensis*, by avoiding a deregulated type of inflammatory response associated to neutrophils that may lead to tissue damage. Thus modulation of TLR9 may be of interest to potentiate the host response against this pathogen.

## Author Summary

*Paracoccidioides brasiliensis* is an etiological agent of paracoccidioidomycosis, one of the most prevalent systemic mycosis in South America, affecting over 10 million people. One of the hallmark features of this fungus is its multinucleated nature, where a single *P. brasiliensis* cell can have from one to over 50 nuclei. Thus, fungal cell death may culminate in the release of large amounts of DNA and in the activation of the host innate immune system via toll like receptor (TLR) 9, a member of the TLR family. In this study, we evaluated the contribution of TLR9 activation during the infection by *P. brasiliensis* using an experimental model of *P. brasiliensis* infection of wild-type and TLR9^−/−^ mice. We found that the lack of this receptor results in tissue damage and increased expression of cytokines, culminating in a higher death rate of TLR9^−/−^ mice early post infection. This was mainly associated to neutrophils, as depletion of these cells, during the first 48 hours post-infection, converted the early response of TLR9^−/−^ mice to infection to that of wild-type mice. Our work provides evidence for the protective effects of TLR9 activation during early stages of *P. brasiliensis* infection, highlighting the relevance of a balanced innate immune response to avoid tissue damage.

## Introduction

*Paracoccidioides brasiliensis* is a causative agent of paracoccidioidomycosis (PCM), one of the most prevalent systemic mycosis in Latin America [1]. One of *P. brasiliensis* biological hallmarks is its particular temperature-dependent morphological dimorphism. This fungus switches from the environmental non-pathogenic mycelial/conidial form at ambient temperatures to the pathogenic multiple budding yeast form, with an high variability of cell sizes, when exposed to temperatures similar to those of the mammalian host [2], [3], [4]. The mechanism of infection by *P. brasiliensis* entails the inhalation of airborne conidia that, when in the lung and exposed to host temperatures, undergo a complex morphological switch to the pathogenic yeast form [1], [5]. PCM is divided in two different forms: the acute or sub-acute form and the chronic form, depending on the natural course of infection and clinical manifestations of the patient [1], [6]. The clinical manifestations rely on the virulence of *P. brasiliensis* infecting strain, the degree and type of immune response triggered, the tissues infected, and importantly, on intrinsic characteristics of the host [7], [8].

Despite the fact that a large number of individuals are exposed to the fungus, only a minority develops the disease, suggesting that, for the majority of the population, a protective immune response is developed [6], [9]. Therefore, the understanding of the protective characteristics of the immune response to *P. brasiliensis* is of interest as it may reveal targets for disease control. The initiation of the immune response relies on the activation of the innate immune system upon recognition of pathogen-associated molecular patterns (PAMPs) [10]. This recognition is mediated by the family of pattern recognition receptors (PRRs) that is composed by a large number of receptors in immune cells [11], [12]. Activation of PRRs culminates with the expression of several immune mediators, including pro- and anti-inflammatory cytokines and also with the activation of a series of microbicidal mechanisms that aim at eliminating the pathogen [13]. The most widely recognized type of PRRs are the toll-like receptors (TLRs) [14]. Over the past few years, several studies have demonstrated a relevant role for TLRs in the recognition of fungal pathogens, such as *P. brasiliensis*, *Candida albicans*, *Aspergillus fumigatus*, and *Cryptococcus neoformans* [15], [16], [17], [18], [19], [20], [21]. The role of MyD88, an adaptor protein used by all TLRs (with the exception of TLR3), during *P. brasiliensis* infection remains controversial, with some authors reporting that this protein is not essential for an effective response against *P. brasiliensis* [22], and others claiming that MyD88 is important for the activation of innate fungicidal mechanisms and for the induction of the effector and regulatory cells of the adaptive immune response [23]. A role for both TLR2 and TLR4 in the recognition and internalization of *P. brasiliensis* has been reported in human monocytes and neutrophils [24]. In a model of experimental PCM, TLR2 deficiency leads to increased Th17 immunity associated with diminished expansion of regulatory T cells and increased lung pathology due to unrestrained inflammatory reactions [25]. In contrast, *P. brasiliensis* recognition by TLR4 leads to an increased production of Th17 cytokines, enhanced pro-inflammatory immunity, and impaired expansion of regulatory T cells, resulting in a more severe form of infection [26]. However, the involvement of TLR9 in *P. brasiliensis* infection has not yet been addressed. Several lines of evidence suggest that TLR9 may play a role in infection by *P. brasiliensis*, similarly to what is already described for other pathogenic fungi, such as *A. fumigatus*, *C. albicans* and *C. neoformans* [27],[28],[29],[30],[31],[32]. Firstly, *P. brasiliensis* DNA is known to have large numbers of CpG motifs [33], the natural ligand to TLR9 [34]. Secondly, due to the fungus multinucleated nature [35], [36], a high amount of DNA is expected to be released upon cell death during infection. Thirdly, previous studies show that, in *in vitro* models, *P. brasiliensis* DNA increases the phagocytic index of macrophages, whereas in *in vivo* models of *P. brasiliensis* infection, TLR9 activation may act as a Th1-promoting adjuvant in a time/concentration dependent-manner [33], [37].

In this study, we investigated the role of TLR9 in the recognition of *P. brasiliensis*, and its influence on the infective process and evolution of the disease. Our results show that TLR9 recognizes *P. brasiliensis*, playing a major regulatory role during early times of *in vivo* infection with its absence making the host more prone to increased liver pathology and premature death, mainly mediated by neutrophils.

## Materials and Methods

### Microorganisms and culture media

The strain ATCC 60855 of *P. brasiliensis* registered at the American Type Culture Collection (Rockville, MD) was used throughout this experiment. Yeast cells were maintained at 37°C by subculturing in brain heart infusion (BHI) (Duchefa) solid media supplemented with 1% glucose and gentamicin (50 µg/mL). For both *in vitro* and *in vivo* assays, yeast cells were grown in BHI liquid medium supplemented with 1% glucose and gentamicin (50 µg/mL) at 37°C with aeration on a mechanical shaker (220 rpm). Cell growth was monitored for 148 h by microscopic counting using a Neubauer's Chamber and cells were collected during the exponential growth phase (72 h of growth, 1.65±0.8×10^7^ cells/mL) for all the experimental assays.

### Ethics statement

This study was approved by the Portuguese national authority for animal experimentation Direção Geral de Veterinária (ID: DGV 594 from 1st June 2010). Animals were kept and handled in accordance with the guidelines for the care and handling of laboratory animals in the Directive 2010/63/EU of the European Parliament and of the Council.

### Mice

Eight-week-old C57BL/6 mice were obtained from Charles River (Barcelona, Spain) and eight-week-old TLR9^−/−^ (generated in a C57BL/6 background) were kindly provided by P. Vieira (Pasteur Institute of Paris, France). Mice were housed under specific-pathogen-free conditions with food and water *ad libitum*.

#### *In vivo* infection

C57BL/6 WT or TLR9^−/−^ mice were infected intravenously (i.v.) with 1×10^6^ *P. brasiliensis* yeast cells grown to the exponential phase in BHI liquid medium. Prior to infection, cells were washed 3 times with lipopolysaccharide (LPS)-free phosphate-buffered saline (PBS) (Gibco), passed through a syringe to eliminate cell clumps, and submitted to Neubauer counting procedures (each mother and bud cells were considered as individual counts). Mice were monitored daily for 32 days. Mice showing severely impaired mobility in the first 48 hours of infection were sacrificed and liver and blood were collected for later analysis.

For the peritoneal cavity model of infection, mice were injected intraperitoneally (i.p.) with 1×10^6^ *P. brasiliensis* yeast cells grown and treated as described above. Mice were sacrificed 6 hours post-infection and, after peritoneal lavage with 4 ml of PBS, total leukocyte number was determined.

### Histological studies

Liver from dying mice were harvested in the time-period comprehending 48 h post-infection, fixed in 3.8% phosphate-buffered formalin and embedded in paraffin. Light-microscopy studies were performed on tissue sections stained with hematoxylin and eosin (HE) as previously described [38]. The histological analysis was performed by the presence of necrotic areas and the type of inflammatory infiltrate (when present) in each field of 10× objective.

### Neutrophil depletion

C57BL/6 WT and TLR9^−/−^ mice were made neutropenic by treatment with the monoclonal antibody (MAb) RB6-8C5, as previously described [39], [40]. Briefly, mice were injected i.v. in the lateral tail vein with 200 mg of MAb RB6-8C5, and 6 h later infected i.v. with 1×10^6^ *P. brasiliensis* yeast cells grown to the exponential phase in BHI liquid medium and treated as indicated before. Mice were monitored as indicated before during the first 2 days of infection.

#### Preparation of fungal DNA

*P. brasiliensis* DNA was extracted from exponentially growing cells. Briefly, cells were disrupted using a mix of lysis buffer (1 mM EDTA, 10 mM Tris–HCl, 1% SDS, 100 mM NaCl) and phenol/chloroform/isoamylalcohol (25∶24∶1). Heat-shock treatment (45 min at 65°C) was performed and the aqueous phase was treated using a column-based method (in-column proteinase K and RNAse treatments were performed). To elute DNA, LPS-free water was used.

### *In vitro* infection

Bone marrow-derived macrophages (BMDMs) from C57BL/6 WT and TLR9^−/−^ mice were prepared as described previously [38], [41], [42]. BMDMs were seeded in 24-well plates at 5×10^5^ cells/well and kept at 37°C and 5% CO_2_ atmosphere. Cells were challenged with 5 µg of *P. brasiliensis* DNA extracted from exponentially growing cells. In other experiments, BMDMs from C57BL/6 WT and TLR9^−/−^ mice seeded as described above were challenged with *P. brasiliensis* yeast cells grown to the exponential phase, with late-stationary growing cultures or with *P. brasiliensis* lysed cells, using a 2∶1 multiplicity of infection (MOI; yeast/BMDMs ratio) for 24 h. Prior to infection, fungal cells were washed 3 times with LPS-free PBS, passed through a syringe to eliminate cell clumps, and submitted to Neubauer counting procedures (mother and bud cells were considered as individual counts). Supernatants from stimulated BMDMs were collected 24 h post-infection and stored at −80°C for later cytokine analysis.

### *P. brasiliensis* phagocytosis assays

BMDMs from C57BL/6 WT and TLR9^−/−^ prepared as previously described [38] were seeded in 24-well plates at 5×10^5^ cells/well, kept at 37°C and 5% CO_2_ atmosphere and infected with *P. brasiliensis* yeast cells grown to the exponential phase using a 2∶1 multiplicity of infection (MOI; yeast/macrophage ratio) for 3 h. The wells were then washed three times with PBS to eliminate non-phagocytized cells, BMDMs were lysed with sterile H_2_O and the remaining phagocytized yeast cells were collected to determine total phagocytosis, using a Neubauer's Chamber. For the experiments, *P. brasiliensis* cells treated with DNase were also used. Briefly, after collecting and washing, *P. brasiliensis* yeast cells grown to the exponential phase were incubated at 37°C for 1 h with 20 µg of DNaseI (Ambion). As control, heat-inactivated DNase (70°C for 1 h) was used.

### ELISA

Cytokine levels were measured in serum collected from infected animals or in supernatants of infected cell cultures by capture enzyme-linked immunosorbent assay (ELISA) (eBioscience). The ELISA procedure was performed according to the manufacturer's protocol, and absorbances were measured with a Bio-Rad 680 Micro-plate Reader.

### Flow cytometry

Quantification of neutrophils and mononuclear cells influx to the peritoneal cavity during the i.p. infection with *P. brasiliensis* was performed by flow cytometry (FCM) on a BD LSR II flow cytometer. Cells collected from the peritoneal cavity were stained, using specific antibodies for CD11b, CD11c, GR-1 and Ly6G to distinguish neutrophils and mononuclear cells populations, and a minimum of 100,000 cells per sample was acquired at low/medium flow rate. Offline data was analyzed with the flow cytometry analysis software package FlowJo 7.6.1.

### Real-time polymerase chain reaction (RT-PCR)

Total RNA (1 µg) was isolated according to TRIzol methodology (Invitrogen). For liver samples, small portions were homogenized in between microscopy slides, suspended in 1 mL of TRIzol reagent and kept at −80°C for later analysis. For the i.p. experiment around 5×10^5^ cells from the peritoneal exudates were used, suspended in 250 uL of TRIzol reagent and stored at −80°C for later analysis. RNA integrity was checked by the presence of clear 18S and 28S rRNA bands in agarose gel electrophoresis. The absence of DNA contamination in the samples was confirmed by the absence of PCR amplification of the ubiquitin gene in the isolated RNA. Total RNA (1 µg) was reverse transcribed using the iScript cDNA Synthesis kit (Bio-Rad) following manufacturer's instructions and 1 µL of cDNA used as a template for real-time quantification using the SsoFast EvaGreen SuperMix (Bio-Rad) following manufacturer's instructions. Real-time quantification was carried out on a CFX96 Real-Time System (Bio-Rad) using threshold cycle (Ct) values for ubiquitin transcripts as the endogenous reference. The primer sequences were designed and synthesized by TIB Mol. Biol. and were as follows: UBQ forward, *TGG CTA TTA ATT ATT CGG TCT GCA T*; UBQ reverse, *GCA AGT GGC TAG AGT GCA GAG TAA*; IL-10 forward, *TTT GAA TTC CCT GGG TGA GAA*; IL-10 reverse, *GCT CCA CTG CCT TGC TCT TAT T*; IL-17 forward, *CTC AGA CTA CCT CAA CCG TTC CA*; IL-17 reverse, *TTC CCT CCG CAT TGA CAC A*; TNF forward, *GCC ACC ACG CTC TTC TGT CT*; TNF reverse, *TGA GGG TCT GGG CCA TAG AAC*; MIP2 forward, *CTC AGT GCT GCA CTG GT*; MIP2 reverse, *AGA GTG GCT ATG ACT TCT GTC T*; IL-6 forward, *TCG TGG AAA TGA GAA AAG AGT TG*; IL-6 reverse, *TAT GCT TAG GCA TAA CGC AC TAG*. All measurements were performed in triplicate. A single melting peak was obtained for each gene analyzed in all samples.

### Statistics

**Figure 1:**
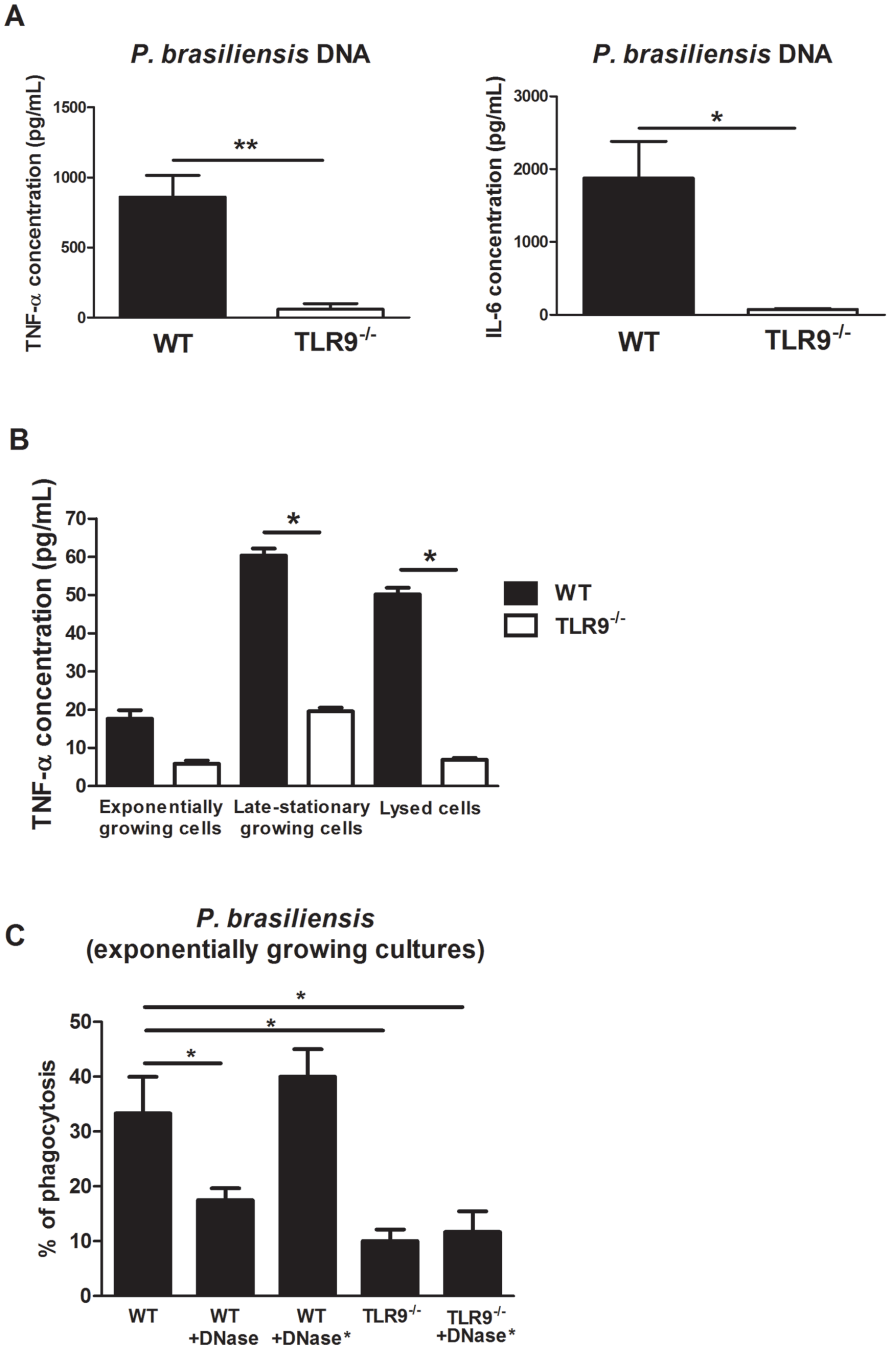
Outcome of *in vitro* stimulation of macrophages from WT and TLR9^−/−^ mice. (A) - Protein levels after stimulation with 5 µg of *P. brasiliensis* DNA for 24 h. Asterisks represent significant differences between WT and TLR9 TLR9^−/−^ mice (**P<0.01, *P<0.05). IL-6 was also found with higher expression levels in dendritic cells from TLR9^−/−^ mice when compared to dendritic cells from WT mice (data not shown) (B) – Protein levels after stimulation with wild-type *P. brasiliensis* ATCC 60855 yeast cells grown either to the exponential or late-stationary phase for 24 h using a MOI of 2∶1, and lysed cells. Asterisks represent significant differences between WT and TLR9^−/−^ mice. IL-6 levels were below detection limits; (C) - Percentage of total phagocytosis of wild-type *P. brasiliensis* ATCC 60855 yeast cells grown to the exponential phase using a MOI of 2∶1. Asterisks represent significant differences between macrophages from WT mice stimulated with *P. brasiliensis* yeast cells and either macrophages from WT mice stimulated with *P. brasiliensis* yeast cells pre-treated with DNase (WT+DNase) (*P<0.05) or macrophages from TLR9^−/−^ mice stimulated with *P. brasiliensis* yeast cells (TLR9^−/−^) (*P<0.05). Pre-treatment of *P. brasiliensis* yeast cells with DNase does not affect phagocytosis by TLR9^−/−^ macrophages (data not shown). When cells were treated with heat inactivated DNase, the phagocytic profile of WT macrophages is restored (WT^−/−^+DNase*), while for TLR9^−/−^ macrophages it is not altered (TLR9^−/−^+DNase*).

**Figure 2:**
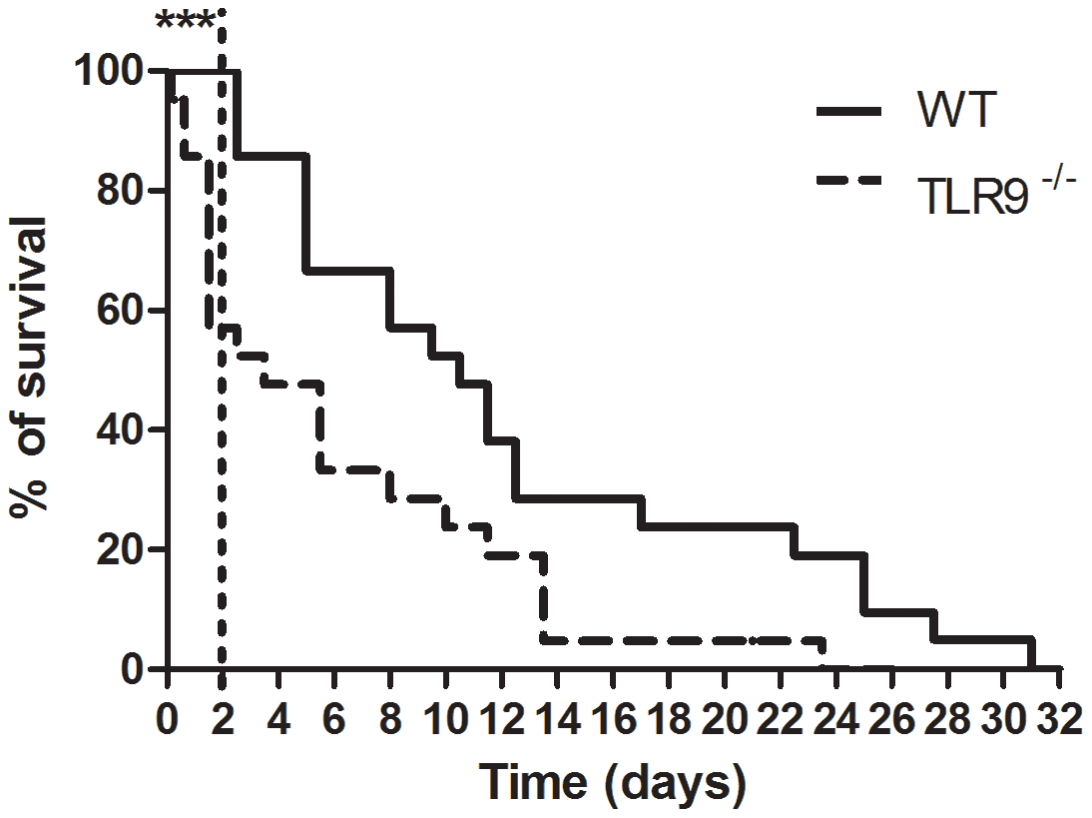
Outcome of *in vivo* intravenous infection of WT and TLR9^−/−^ mice with *P. brasiliensis*. Representative survival curves using the Kaplan-Meier estimator of an experimental intravenous infection carried out in WT and TLR9^−/−^ C57BL/6 mice (n = 21) with 1×10^6^ wild-type *P. brasiliensis* ATCC 60855 yeast cells grown to the exponential phase. The estimated mean survival for WT mice is 13.6±2.0, while for TLR9^−/−^ mice is 6.8±1.4 (Gehan-Breslow-Wilcoxon test was applied). Data is expressed as percentage of live animals. The observed differences during the first 48 h post- infection were statistically significant (***P<0.001).

Data is reported as the mean ± standard error of the mean (SEM) and all assays were repeated at least three times. All statistical analysis was performed using the GraphPad Prism Software version 5.01. For the experiments comparing two groups (see Fig. 1A and B, 4 and 5), a two-tailed unpaired Student *t* test was performed. Welch's correction was applied when making multiple comparisons. The One Way ANOVA test was performed in data presented in Fig. 1C using Turkey's multiple comparison post-test. The survival curves, representative of three independent experimental infections (Fig. 2, n = 21 mice), are represented using the Kaplan-Meier estimator, and Gehan-Breslow-Wilcoxon test was applied. For all data analysis statistical significance was considered at the level of 0.05 (2-tailed, 95% confidence interval).

## Results

### Triggering of TLR9 by *P. brasiliensis* modulates macrophage cytokine production and phagocytosis

Since previous studies have reported the recognition of fungal DNA by TLR9 [43], we questioned if *P. brasiliensis* triggers TLR9-mediated responses in macrophages. For that, we stimulated BMDMs generated from wild-type (WT) and TLR9^−/−^ mice with purified *P. brasiliensis* DNA. Our results showed that purified *P. brasiliensis* DNA induced the secretion of TNF-α and IL-6 by BMDMs in a TLR9 dependent way (Fig. 1A). Next, we tested if TLR9 could also recognize *P. brasiliensis* DNA in the yeast cellular context and whether this depended on the cellular physiological stage and integrity. We stimulated WT and TLR9^−/−^ BMDM with either *P. brasiliensis* cells from exponential or late-stationary growing cultures or with lysed cells. We found that BMDMs stimulated with *P. brasiliensis* yeast-form produced TNF-α in a TLR9 dependent manner (Fig. 1B), although the response was higher for late-stationary growing and lysed cells.

In addition to cytokine expression, TLR triggering also associates with the onset of phagocytosis [44], [45]. We next investigated if TLR9 activation by the yeast-form of *P. brasiliensis* impacted phagocytosis. The percentage of phagocytosis of *P. brasiliensis* by BMDMs was significantly reduced when *P. brasiliensis* yeast cells were treated with DNase or when TLR9^−/−^ BMDMs were used (Fig. 1C). When heat inactivated DNase was used, the percentage of phagocytosis by WT macrophages was similar to that observed when no treatment was performed, whereas TLR9*^−/−^* macrophages maintained the phagocytic profile, indicating that DNase is not interfering with phagocytosis. Thus, our data indicate that TLR9 activation is required for maximal *P. brasilisensis* phagocytosis.

### TLR9 has a protective role in the early phase of *P. brasiliensis* infection

Given the *in vitro* impact of *P. brasiliensis* recognition by TLR9, we next sought to investigate a role for this receptor during the course of an *in vivo* experimental infection. WT and TLR9^−/−^ mice were intravenously infected with *P. brasiliensis* and the survival rates followed over time. We found that 24 days post-infection 100% of TLR9^−/−^ mice had succumbed, whereas for WT mice this was only observed 31 days post-infection (Fig. 2). Furthermore, the estimated mean survival for WT mice was of 13.6±2.0 days, while for TLR9^−/−^ mice it was reduced to 6.8±1.4 days (p<0.001; Fig. 2). Even more striking was the observation that during the first 48 h post-infection, TLR9^−/−^ infected mice showed severe impaired mobility, with a significant number of animals being humanely sacrificed as a result. As shown in Fig. 2, during this early period the mortality of the TLR9^−/−^ mice was approximately 43%, while WT mice showed no signs of disease. Thus, our data highlight an unexpected protective role of TLR9 during the early phases of *P. brasiliensis* infection.

### Absence of TLR9 associated with increased immunopathology early during *P. brasiliensis* infection

**Figure 3:**
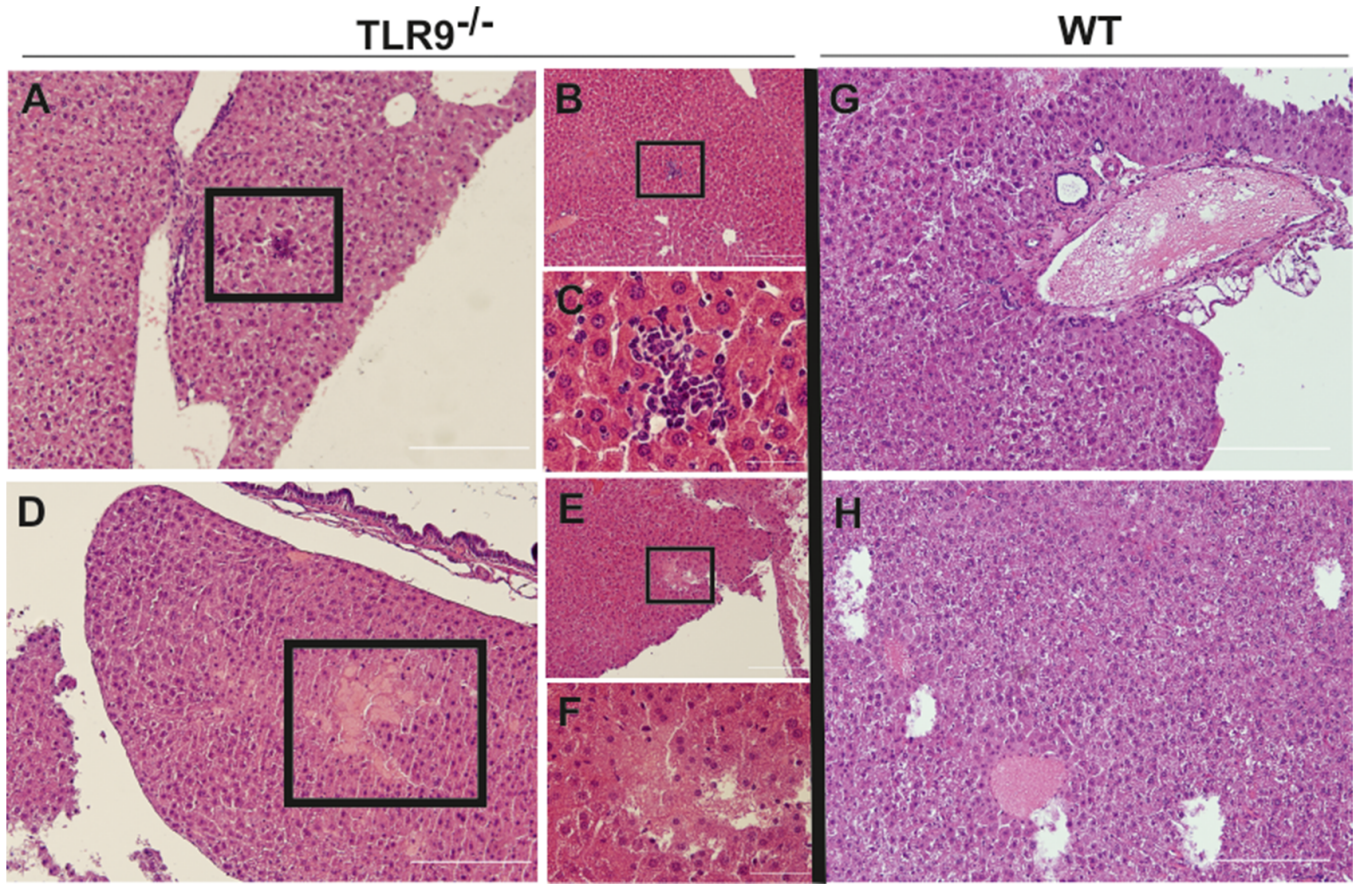
Histological analysis of liver from TLR9^−/−^ and WT mice intravenously infected with *P. brasiliensis*. Histological sections of liver stained with HE from TLR9^−/−^ C57BL/6 mice (A to F) and WT C57BL/6 mice (G and H) intravenously infected with 1×10^6^ wild-type *P. brasiliensis* ATCC 60855 yeast cells grown to the exponential phase. Magnification: ×10 (A, B, D, E, G and H); ×40 (F) and ×60 (C). Squares represent either tissue with inflammatory infiltrates (A and B) or with necrotic areas (D and E). Figures C and F represent magnifications of B and E, respectively. Results are from one representative experiment of two independent experiments. White bars represent 200 µm.

**Figure 4:**
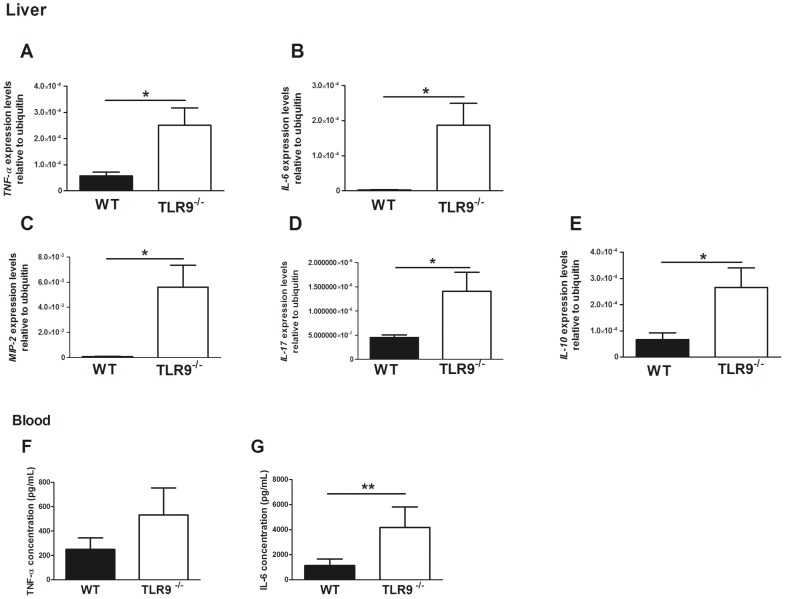
Cytokine levels detected upon intravenous infection of WT and TLR9^−/−^ mice with *P. brasiliensis*. Levels of cytokines detected upon intravenous infection of WT and TLR9^−/−^ C57BL/6 mice (n = 21) with 1×10^6^ wild-type *P. brasiliensis* ATCC 60855 yeast cells grown to the exponential phase. Samples were taken from dying mice during the first 48 h post-infection. As control, samples from the same number of healthy WT mice (n = 9) were collected. (A–E) – Expression profile of TNF-α, IL-6, MIP-2, IL-17, and IL-10 at mRNA level in liver from the mice. Asterisks represent significant differences between WT and TLR9^−/−^ mice (*P<0.05); (F and G) - Protein levels of TNF-α and IL-6 detected via ELISA in the blood serum of challenged mice. Asterisks represent significant differences between WT and TLR9^−/−^ mice (**P<0.01). There is also a trend towards higher TNF-α levels in TLR9^−/−^ mice, though no statistically significant differences were detected. Bars represent means and standard deviations.

In view of the key protective role observed for TLR9 during the early stages of *P. brasiliensis* infection, we next investigated several parameters that could be associated with the premature death of TLR9^−/−^- infected animals. Since during the initial period of infection (the first 48 h) differences in the lungs of infected animals are difficult to assess, we performed a histological analysis of the liver of the infected animals. We found that whereas the livers of infected WT animals showed a normal structure, those of TLR9^−/−^ hosts presented small areas of granulocytes/neutrophil infiltrates and of necrosis (Fig. 3). To dissect further if the histological differences observed were associated to the intensity of the immune response between WT and TLR9^−/−^ animals, we assessed cytokine expression in the liver and blood of infected animals. In the liver, the expression of both pro- and anti-inflammatory cytokines, namely TNF-α, IL-6 and IL-10, was increased in the absence of TLR9 (Fig. 4A–E). Likewise, a significant increase of circulating levels of IL-6 was observed in TLR9^−/−^-infected mice (Fig. 4G). Moreover, a trend towards higher levels of circulating TNF-α was detected (Fig. 4F) whereas those of IL-10 were below detection limit (data not shown).

**Figure 5:**
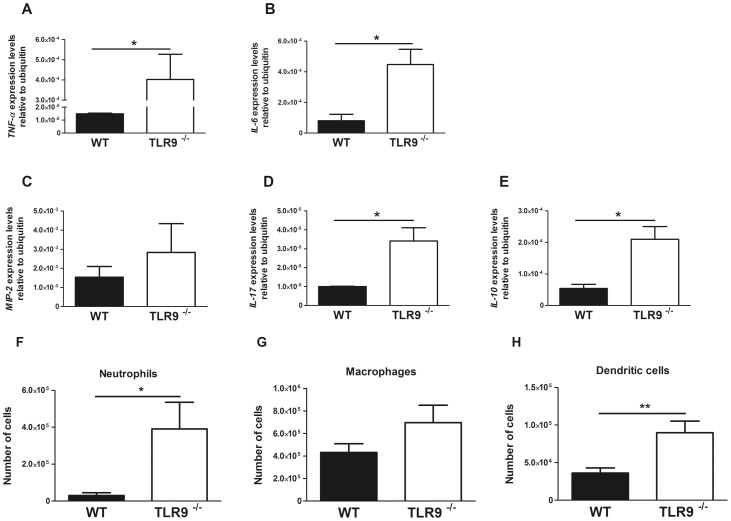
Outcome of intraperitoneal infection of WT and TLR9^−/−^ mice with ***P.*** **brasiliensis**** **.** **** Intraperitoneal infection of WT and TLR9^−/−^ C57BL/6 mice (n = 10) with 1×10^6^ *P. brasiliensis* yeast cells grown to the exponential phase. (A–E) - Expression profile of TNF-α, IL-6, MIP-2, IL-17, and IL-10 at mRNA level in cells collected from the peritoneal cavity of WT and TLR9^−/−^ mice. Peritoneal lavages were performed 6 h after infection and gene expression levels were accessed by RT-PCR. Asterisks represent significant differences between WT and TLR9^−/−^ mice (*P<0.05); (F–H) - Influx of neutrophils and mononuclear cells to peritoneal cavities of WT and TLR9^−/−^ mice. Peritoneal lavages were performed 6 h after infection, and total and differential leukocyte counts were done. An average of 2.1×10^6^±1.4×10^6^ cells was collected. Asterisks represent significant differences between WT and TLR9^−/−^ mice (*P<0.05, **P<0.01). Bars represent means and standard deviations.

To further validate if the absence of TLR9 correlated with an exacerbated immune response to *P. brasiliensis*, and to study differential patterns of cellular recruitment in WT versus TLR9^−/−^ mice, we used a model of i.p. infection The analysis of the peritoneal exudates revealed that the cytokine expression was increased in the absence of TLR9 (Fig. 5A–E). Of notice, a marked increased expression of both MIP-2 and IL-17, known to be associated with neutrophil recruitment [46], [47], [48], was found in the absence of TLR9 (Fig. 5C, D). Consistently, in TLR9^−/−^ mice we found an increased recruitment to the peritoneal cavity of both mononuclear cells (macrophages and dendritic cells) and neutrophils, with a special significance of the latter ones (Fig. 5F–H). Thus, the data obtained for peritoneal infection recapitulates that obtained for intravenous infection.

### Neutrophilia mediates the early death of *P. brasiliensis*-infected TLR9^−/−^ mice

**Figure 6:**
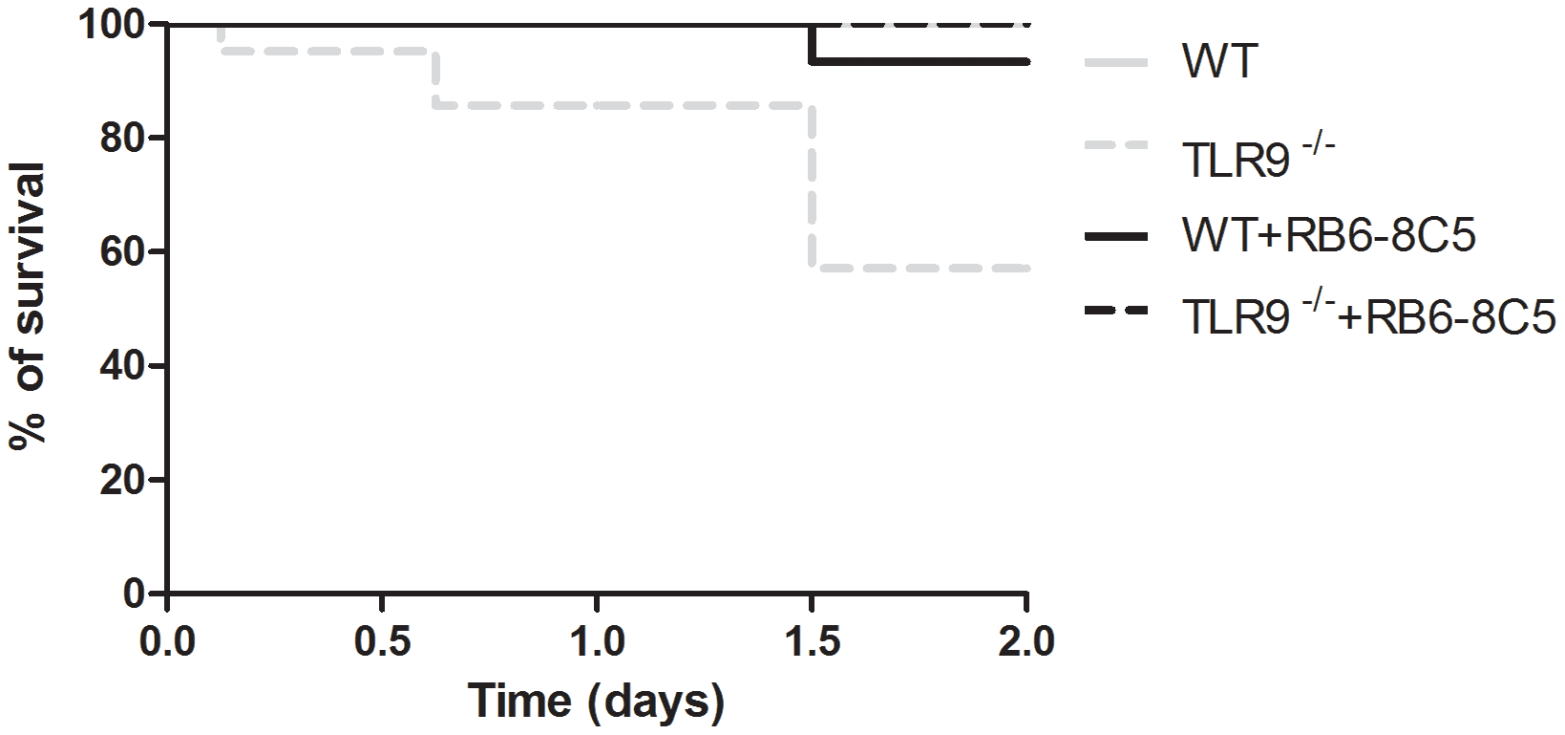
Outcome of *in vivo* intravenous infection of WT and TLR9^−/−^ neutropenic mice with *P. brasiliensis*. Representative survival curves of an experimental intravenous infection carried out in WT and TLR9^−/−^ C57BL/6 mice (n = 21) with 1×10^6^ wild-type *P. brasiliensis* ATCC 60855 yeast cells grown to the exponential phase. Mice were treated i.v. with the neutrophil-depleting MAb RB6-8C5 6 h pre-infection. Grey lines represent the survival curve of the experimental intravenous infections carried out in WT and TLR9^−/−^ C57BL/6 mice (n = 21) with 1×10^6^ wild-type *P. brasiliensis* ATCC 60855 yeast cells grown to the exponential phase.

Considering the high influx of neutrophils to the peritoneal cavity of TLR9^−/−^ mice, which correlated with the high expression of MIP-2 and IL-17 found in the liver and peritoneal cavity, we next evaluated if this could be the detrimental factor during the infectious process of TLR9^−/−^ hosts. For this purpose, we depleted neutrophils in WT and TLR9^−/−^ mice prior to infection with *P. brasiliensis*, using a specific monoclonal antibody (MAb RB6-8C5). I.p. injection of MAb RB6-8C5 abrogated neutrophils from 6 h to up to 48 h post-injection (data not shown, [40]). As shown in figure 6, depletion of neutrophils reverted the highly susceptible phenotype observed in TLR9^−/−^ mice during the first two days of infection. Depletion of neutrophils in WT mice had no influence on the mice survival during the first 48 h post-infection (Fig. 6). Therefore, a high neutrophil recruitment to the site of infection appears to be responsible for the death observed in the absence of TLR9 during the early stages of *P. brasiliensis* infection.

## Discussion

Since the discovery of TLRs and their major role in host recognition of conserved molecular structures from microorganisms, particularly those of invading pathogens, enormous advances have been made in comprehending how the immune system responds to pathogenic organisms. It is well established that the first phase of an immune response involves innate immune mechanisms [49], namely those triggered by TLR activation [10], [50], [51]. These receptors are present in neutrophils, monocytes and macrophages, cells that besides their phagocytic activity are crucial for the signaling and amplification of the response against the pathogen [43].

The knowledge on PRR recognition and activation of an efficient immune response against *P. brasiliensis* has progressively increased, mainly in what concerns TLR2, TLR4, and Dectin-1 [24], [52]. However, a role for TLR9 during *P. brasiliensis* infection has only been addressed in the context of vaccination [37], despite the evidences for the involvement of this TLR in other fungal infections [27], [28], [30], [53]. Considering the high DNA content and number of unmethylated CpG oligonucleotides of this multinucleated fungus [33], a role for this receptor during *P. brasiliensis* infection is likely. We herein demonstrate that *P. brasiliensis* purified DNA activated TLR9 in macrophages, leading to the expression of cytokines. This is in line with previous studies showing that fungal DNA is recognized by TLR9. *A. fumigatus* DNA stimulates the production of pro-inflammatory cytokines in mouse and human dendritic cells [30]. Similarly, murine dendritic cells express IL-12p40 and CD40 upon stimulation with DNA from *C. neoformans* [27]. Human monocytes and macrophages from TLR9^−/−^ mice were described to produce less IL-10 than cells from control mice when stimulated with *C. albicans* [28].

Despite TLR9 activation by purified *P. brasiliensis* DNA, upon macrophage stimulation with *P. brasiliensis* yeast cells grown to the exponential phase, TLR9 was activated in a lesser extent. This finding is likely due to the low exposure of DNA in this case, as when yeast cells on late stationary phase or lysed cultures were used, TLR9 recognized *P. brasiliensis* more prominently, leading to the production of pro-inflammatory cytokines by macrophages. Even though intact *P. brasiliensis* yeast cells did not fully activate TLR9 to induce the production of pro-inflammatory cytokines, in the absence of this receptor macrophages showed a decreased ability to phagocyte *P. brasiliensis*. Our findings are in line with previous studies showing that *P. brasiliensis* DNA activates macrophages, promoting their capacity to phagocyte *P. brasiliensis* [33]. Altogether, our data indicate that TLR9 triggering affected the overall responsiveness of macrophages to *P. brasiliensis*. Furthermore, during infection, the release of DNA from *P. brasiliensis* is expected to occur following fungal cell death, thus suggesting that TLR9 activation *in vivo* is very likely to happen.

To assess the role of TLR9 in *P. brasiliensis* infections, we used *in vivo* models of infection. As our results show, TLR9 is crucial for mice survival in early times of infection (first 48 h). Remarkably, we found that, in mice showing severe signs of disease, lack of TLR9 increased the expression of pro-inflammatory cytokines in the liver. Earlier studies on immune responses to other organisms have revealed that an excessive inflammatory response, mainly due to high levels of TNF, can lead to premature death of the host cells [54], [55], [56], [57]. In the context of *P. brasiliensis* infection, it was demonstrated that an excessive inflammatory response may be detrimental rather than protective. A more severe disease development in mice susceptible to *P. brasiliensis* was associated with the presence of increased IL-12 and IFN-γ levels in the lungs, suggesting that the production of pro-inflammatory mediators does not always correlate with immunoprotection [58]. In addition to high TNF expression, we also found increased expression of MIP-2 and IL-17 in the liver and cells from the peritoneal cavity of *P. brasiliensis*-infected TLR9^−/−^ mice. A detrimental role of IL-17 during *P. brasiliensis* infection was previously described as TLR2 and TLR4 deficiency associate with an increase of Th17 responses, lung pathology and more severe forms of infection [25], [26]. Both IL-17 and MIP-2 have been previously associated with neutrophil recruitment [47], [48], [59], [60]. In line with this, we observed a high influx of granulocytes/neutrophils into the peritoneal cavity of TLR9^−/−^ i.p. infected animals. This enhanced neutrophil recruitment could thus be contributing to the detrimental response observed in *P. brasiliensis*-infected TLR9^−/−^ animals. Indeed, upon transient neutrophil depletion, TLR9^−/−^ mice survived during the first 48 hours post-infection, resembling the phenotype of WT mice. Therefore, the susceptibility profile observed for TLR9^−/−^ mice early after infection with *P. brasiliensis* likely associates with an exacerbated neutrophil recruitment to the site of infection and/or with a particular detrimental phenotype of neutrophils. Although neutrophils are crucial during acute inflammatory response and subsequent resolution of fungal infection, in some situations, due to excessive release of oxidants and proteases, these cells may be responsible for injury to organs and fungal sepsis [61], [62]. In addition to fungal infection, neutrophilia can also be harmful to the host in the context of other infections, such as *M. tuberculosis* and *P. aeruginosa* [63], [64], [65], [66].

Our data implicating TLR9 in the phagocytic activity of macrophages raises the hypothesis that, in the absence of TLR9, less *P. brasiliensis* cells are phagocytized. Thus, in the absence of this receptor, the extracellular *P. brasiliensis* cells may contribute to an exacerbated recruitment of neutrophils, which can result in a deregulated immune response. Since a significantly higher recruitment of dendritic cells and neutrophils is observed upon infection of TLR9^−/−^ hosts, it is possible that the higher cytokine expression observed in this scenario results from the fact that more producing cells are present. Therefore, a fine tuned balance is required during infection with *P. brasiliensis*, in order to protect the host from infection. Several mechanisms must operate to achieve this balance, as is the case reported for TLR2/TLR4 activation [25]. It is also important to refer that *P. brasiliensis* can be triggering TLR9-independent mechanisms in macrophages, as supported in the literature [67], [68]. One cannot rule out the hypothesis of a parallel activation of other receptors together with TLR9. Several studies refer that Dectin-1 interaction with TLR9 results in a sinergistyc induction of IL-10, TNF-α, IL-2, IL-6 and IL-23 and down-regulation of IL-12 [69], [70]. Studies with *A. fumigatus* reported a link between TLR2-mediated recognition and the phagocytic response [71], whereas internalization of TLR2 with *A. fumigatus* phagossome was demonstrated [72]. As it has been shown for TLR2/TLR4, it is also possible that TLR9 signaling is involved in the instruction of appropriate regulatory T cell responses, a hypothesis currently under investigation.

Overall, this study highlights the relevant role of TLR9/neutrophils for the nature of the immune response to *P. brasiliensis*, and paves the way to the development of new preventive/therapeutical strategies, as resistance patterns of the host can be of great value on the comprehension of susceptibility and pathogenesis of *P. brasiliensis* infections.
